# Contact tracing for vancomycin-resistant *Enterococcus faecium* (VRE): evaluation of the Dutch policy of quintuple screening cultures

**DOI:** 10.1007/s10096-023-04632-7

**Published:** 2023-06-23

**Authors:** Linda J. Wammes, Anne F. Voor in ’t holt, Corné H.W. Klaassen, Margreet C. Vos, Nelianne J. Verkaik, Juliëtte A. Severin

**Affiliations:** 1 Department of Medical Microbiology and Infectious Diseases, Erasmus MC University Medical Center, P.O. Box 2040, 3000 CA Rotterdam, The Netherlands; 2 Present address: Department of Medical Microbiology, LUMC Center for Infectious Diseases, Leiden University Medical Center, Leiden, The Netherlands

**Keywords:** Vancomycin resistance, *Enterococcus faecium*, Infection control, Contact tracing, Policy, Outbreaks

## Abstract

Detection of vancomycin-resistant *Enterococcus faecium* (VRE) is hampered by low sensitivity of rectal swab cultures. This study aimed to define the number of screening cultures needed to increase sensitivity to detect VRE transmission, and to determine time from presumed exposure to detectable colonization. In a tertiary care setting, we retrospectively analyzed data from 9 VRE outbreaks. As a proxy or estimation for time to detectable colonization, the time between first positive culture of the presumed index patient and that of their contacts was determined. Only 64% of secondary cases were positive in the first out of five cultures. By using the first three out of five rectal swabs, 89% (95%CI: 78–95%) of all secondary cases would have been identified. The median number of days between the positive culture of the index patient and the first positive culture of secondary cases was 9 days. Eleven percent of secondary cases would have been missed if only three rectal samples would have been obtained. Furthermore, our results show that one or more rectal swabs taken around day 9 after presumed exposure should at least be included in the screening approach. In our setting, obtaining a fourth and a fifth rectal swab showed a relevant additional value compared to only one to three swabs. Our findings are useful for determining the most effective VRE contact tracing approach to prevent transmission.

**Supplementary Information:**

The online version contains supplementary material available at 10.1007/s10096-023-04632-7.

## Introduction

Vancomycin-resistant *Enterococcus faecium* (VRE) is a microorganism associated with hospital acquired infections (HAI) and colonization. Worldwide, the prevalence of VRE increases, and combined with its ability to survive in the hospital environment despite cleaning and disinfection this is a microorganism of concern [1]. Although its pathogenicity seems to be low for most patients, immunocompromised patients have an increased risk to develop VRE infections [2, 3]. Case fatality rates of VRE infections have been shown to be highest in these patients, who are mainly admitted to hematology and oncology departments [4]. A meta-analysis showed that most risk factors for VRE acquisition, including prior vancomycin administration and use of invasive medical devices, are linked to these same patient groups [2]. Additionally, duration of intensive care unit (ICU) stay was shown to be an independent risk factor for new VRE acquisitions [5]. Because its high tenacity and resistance to disinfection procedures, early detection of patients who are colonized or infected with VRE is important to prevent transmission to other patients and to the hospital environment [1, 6, 7].

Control of nosocomial transmission and halting VRE outbreaks is therefore necessary, but often challenging because there are difficulties in detecting VRE carriage [1, 8]. Sensitivity of a single rectal swab was estimated at around 40 to 80%, with increasing sensitivity when taking multiple swabs [7–9]. Therefore, the Dutch guideline recommends three to five rectal swabs, with each swab taken on a separate day and the last swab taken at least 7 days after the last exposure, as the most optimal approach to detect VRE carriers [10]. This is based on the supposedly intermittent shedding pattern of VRE; however, evidence for this approach is limited. The limited evidence and lack of consensus resulted in differences in infection prevention and control (IPC) strategies in hospitals within and between countries [1, 11]. Therefore, the aim of this current study was to determine the number of screening cultures needed to detect VRE carriers in outbreak settings, in order to determine the increase in sensitivity of any additional culture with a maximum of five. Furthermore, we aimed to determine the time from presumed exposure to detectable colonization, by studying the time interval between the sample date of the positive culture of the first detected patient with VRE and that of secondary cases.

## Methods

### Study design and setting

This retrospective observational study was conducted in the Erasmus MC University Medical Center (Erasmus MC), a tertiary care center in Rotterdam, the Netherlands, from January 2010 until January 2018. During the study period, this hospital consisted of 1200 beds, organized into 48 departments. The adult ICU department comprised of three high-level ICU wards; each ward consisting of only single-occupancy rooms. Permission for the use of these data was obtained from the medical ethical research committee from the Erasmus MC (MEC-2015-306). Participation of patients is authorized through passive informed consent via electronic patient charts. Eligible patients were cross-checked with the opt-out list. Patients who did not allow that their data were to be used for research were not included in the study population.

### Study population, definitions, and culture data

Data on positive VRE cultures from patients of all ages were extracted from our laboratory information system for the period January 1, 2010 until January 31, 2018. From these patients, additional data were collected, such as history of hospital admission(s), and data on VRE cultures (e.g., date of sampling, *van* gene); including positive clinical cultures and all screening cultures (i.e., positive and negative). In our hospital, screening cultures to detect VRE were taken from patients who were admitted in a hospital abroad <2 months ago or underwent surgery in a hospital abroad, or from patients who were in a contact tracing around a positive index patient. In the Erasmus MC, contact tracing for VRE after an unexpected finding of VRE was common practice since 2008. Contact tracing methods differed during the study period. A summary can be found in Online Resource 1. Additionally, reports from the Unit Infection Prevention of the department of Medical Microbiology and Infectious Diseases containing information about index patients and secondary cases were also used.

A patient was defined as index patient when identified as positive for VRE in a culture and when this finding initiated a contact tracing. Secondary cases were defined as patients found positive in screening cultures resulting from contact tracing. The screening policy during the study period was to obtain a minimum of five rectal swabs on consecutive days, as soon as the index patient was detected and contact patients were identified and informed. Index patients (i.e., first VRE finding in a clinical sample), patients with a sample taken for screening purposes other than contact tracing (i.e., admitted in a hospital abroad), and patients with a VRE strain that was different than the strain from the index patient (i.e., *vanA*/*vanB* mismatch) were excluded. Furthermore, patients reported to be VRE-positive in other hospitals prior to admission at the Erasmus MC were excluded.

### Laboratory methods

A positive VRE culture was defined as growth of *Enterococcus faecium* in a culture of any specimen, with phenotypic resistance to vancomycin (i.e., minimal inhibitory concentration [MIC] for vancomycin of >4 mg/L) and/or presence of the *vanA* or *vanB* gene confirmed by PCR on the isolate.

During the study period, the protocol for VRE screening slightly changed (Online Resource 2). An enrichment broth was used throughout the whole study period.

### Time needed to detect colonization

To estimate the time needed after which VRE colonization in secondary cases could be detected, data from IPC patient reports were used. As a proxy for time needed to detect VRE transmission (i.e., detectable VRE colonization in secondary cases after exposure), we calculated the number of days between the sample date of the positive VRE culture of the index patient and the sample date of the first positive VRE culture of the secondary case. This analysis was performed only if detailed admission data (e.g., room numbers during hospital stay) were available.

### Statistical analysis

Data were plotted in Microsoft Excel or GraphPad Prism. IBM Statistical Package for the Social Sciences Solutions (SPSS) version 25 (IBM Corp., Armonk, New York, USA) was used for all analyses.

## Results

### Characteristics of the study population

**Fig. 1:**
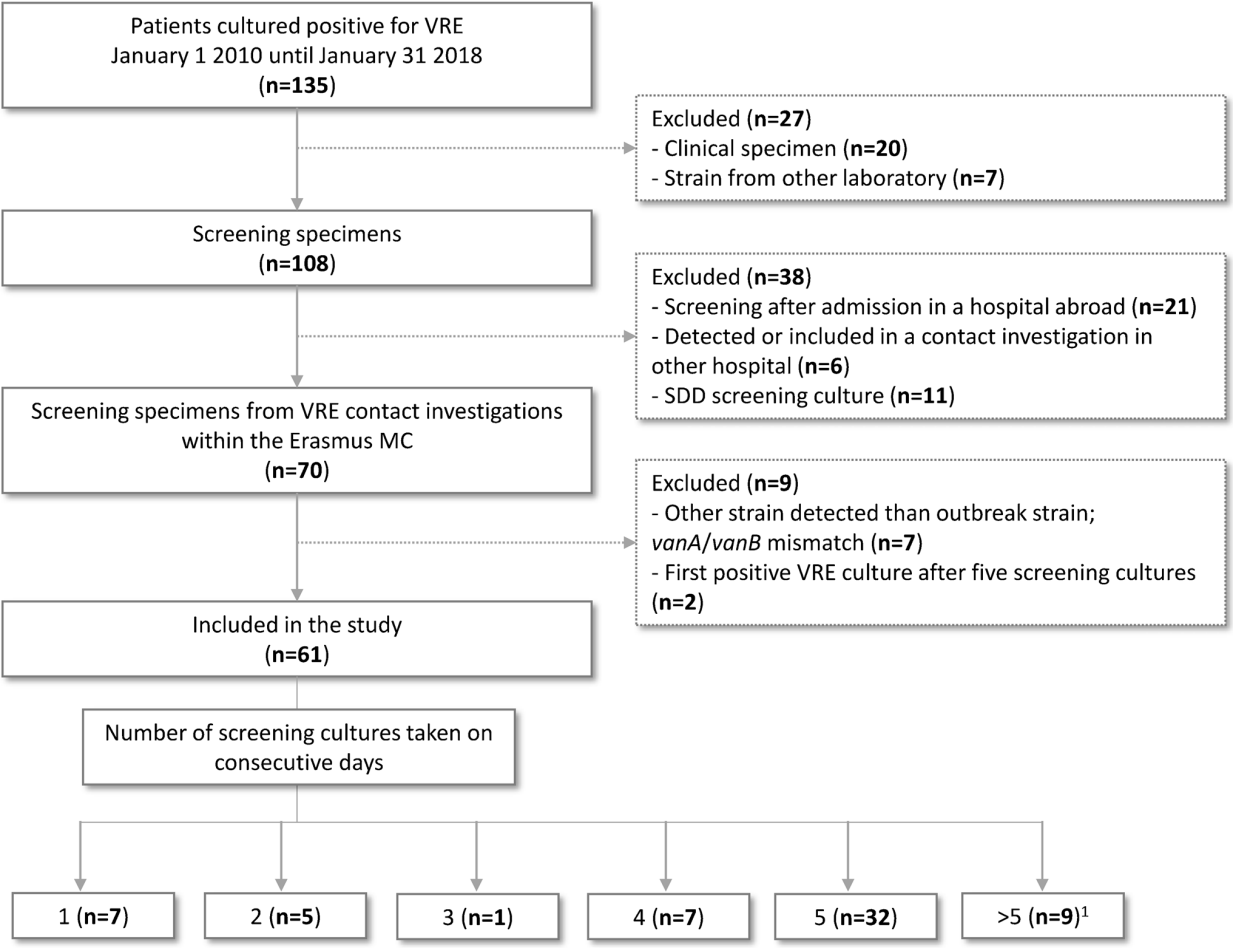
Flow diagram of patient inclusion. Erasmus MC, Erasmus MC University Medical Center; VRE, vancomycin-resistant *Enterococcus faecium*; SDD, selective digestive tract decontamination [12]. ^1^Patients cultured positive in screening cultures 1–5, however, more than five screening cultures were available

Between January 2010 and January 2018, VRE was found in cultures from 135 patients. To identify the secondary cases, index patients, and VRE carriers first detected in other laboratories were excluded (Fig. 1). Additionally, screening cultures obtained for other purposes than contact tracing were excluded (Fig. 1). The remaining 70 VRE-positive isolates belonged to patients defined as contacts and therefore part of contact tracing in our hospital. Since seven patients carried a VRE strain that was different than the index patient, these were excluded (i.e., *vanA*/*vanB* mismatch). Subsequently, two patients were first found positive in the sixth or eighth culture, which was outside the scope of our analysis of five rectal swabs. Finally, a total of 61 patients were included in this study (Fig. 1).

Of these 61 patients, 37 (61%) were male, and the median age was 61 years (range 11–84 years old). Nine (15%) and four (7%) patients were screened during or after admission on ICU and hematology wards, respectively. Most patients (*n*=35, 57%) were screened during outbreaks at one of the surgical departments in 2015 (Online Resource 3). During the study period, nine outbreaks were documented, with an extent ranging from 2 to more than 30 patients in each outbreak (Online Resource 3).

### Number of screening cultures needed to detect VRE transmission

To assess the number of cultures needed to detect VRE carriage, the 61 secondary cases were evaluated (overview in Online Resource 4). For 41 patients (67.2%), five screening cultures were obtained on five consecutive days (Fig. 1). Twenty patients had 1 to 4 cultures taken (32.8%), of which seven patients were screened only once, most of whom early in the study period when obtaining five cultures was not routine and established practice yet (Fig. 1, Online Resource 4).

**Fig. 2:**
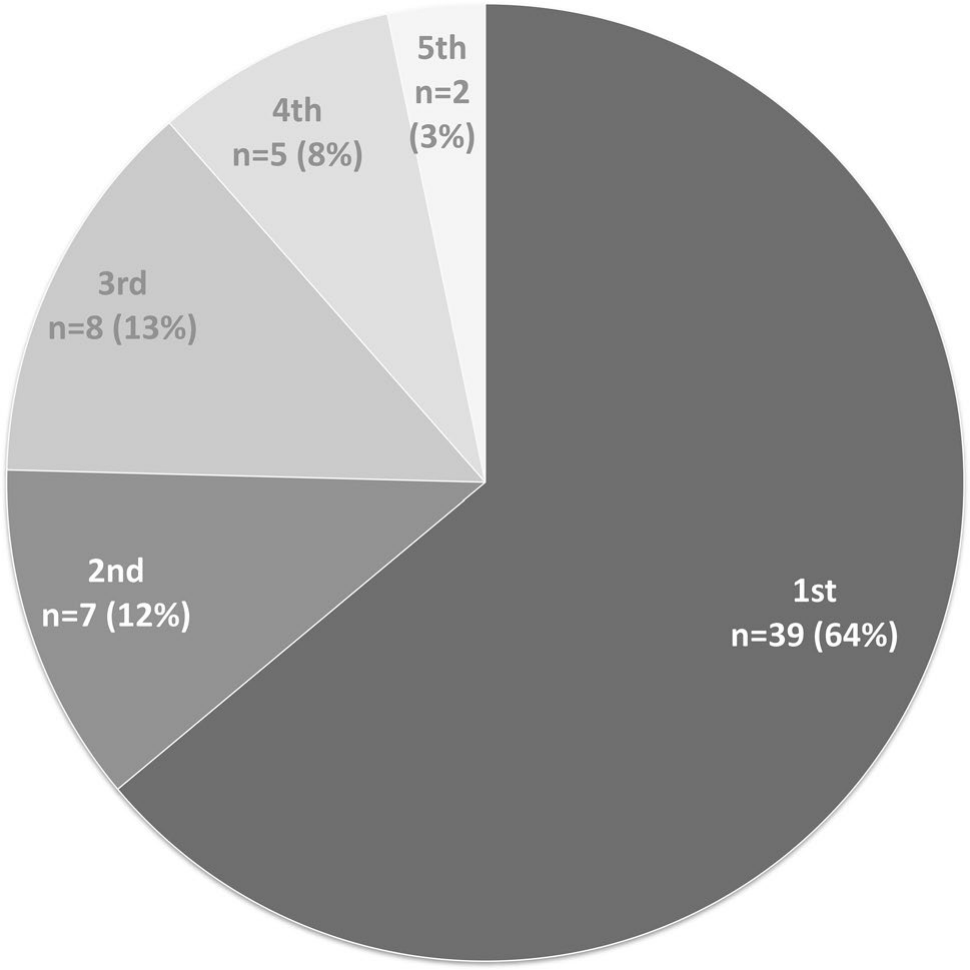
The numbers (*n*) and proportions (%) of patients first identified as positive for VRE in the culture of the first, second, third, fourth, or fifth rectal swab are depicted for in total 61 secondary cases identified in VRE contact tracing

Of the 61 secondary cases, 39 patients (63.9%) were positive for VRE in the first obtained rectum swab (Fig. 2). The second or third culture as first positive was observed in seven (11.5%) and eight (13.1%) patients, respectively. However, five patients (8.2%) were found positive for the first time in the fourth culture and two patients (3.3%) in the fifth culture (Fig. 2). Overall, 88.5% (95% confidence interval 77.8–95.3%) of patients (*n*=54) included in contact tracing were positive for VRE in the culture from the first, second, or third obtained rectal swab. When four or more swabs were obtained (which was the case for 48 out of 61 patients; 78.7%), seven (out of 61; 11.5%) additional VRE carriers were identified. After first being cultured positive, 25 out of 61 patients (41.0%) had one or more consecutive negative screening cultures (Online Resource 4).

### Time to detect VRE transmission

For 53 out of 61 patients positive for VRE in contact tracing (86.9%), the probable index patient could be identified based on concurrent stay on the same ward and agreement in presence of *vanA* or *vanB* gene. For two out of 53 patients, the probable index patient was identified in another hospital; these patients were therefore excluded for further analysis because of missing data. Therefore, the proxy parameter was available for 51 out of 61 patients (83.6%).

Out of 51 contact patients, seven (13.7%) were admitted at or after the date the index patient was identified as VRE-positive (median= 3 days, ranging from 0 to 6 days). This is possible because the date of identification of the index patient is the sample date, not taking the laboratory work into account until the patient is known as being positive, cared for with contact precautions, and thus the contact tracing is initiated. The remaining 44 patients were admitted at the time of identification of the index patient for a median of 17 days (ranging from 1 to 86 days). At the time of identification of the contact patients, contact patients were admitted together with the index patient for a median of 24 days, (ranging from 3–74).

**Fig. 3:**
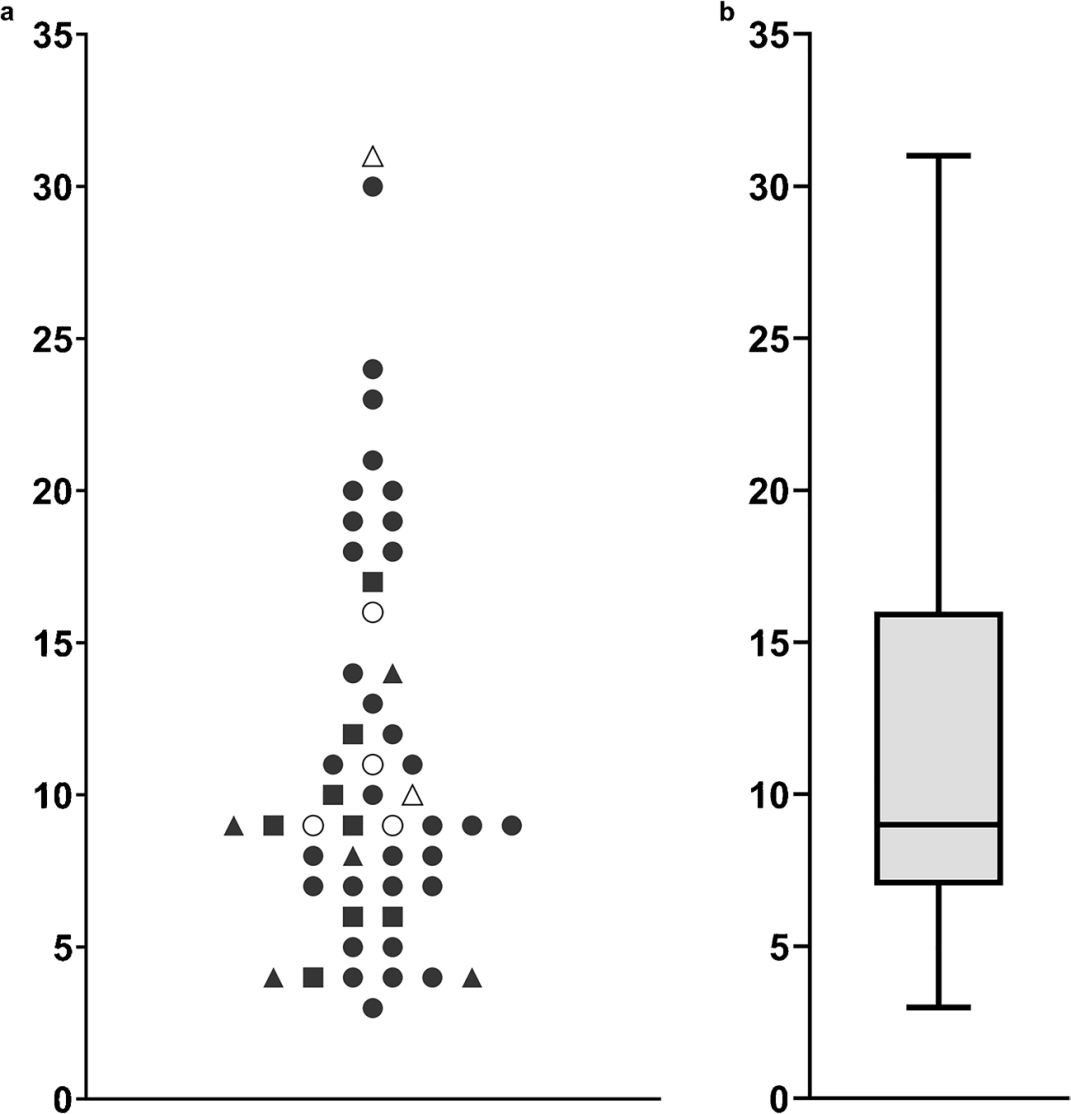
*Y*-axis: the number of days between the sample date of the first positive culture of the index patient and the sample date of the first VRE-positive screening culture of the secondary case. **a** Number of days between sample date of the first positive vancomycin-resistant *Enterococcus faecium* (VRE) culture of the index patient and of the 51 secondary cases. Data were categorized for the consecutive swab number in which VRE was first detected; the symbols represent the first positive culture in a screening set. For cases positive in first (circle), second (triangle), or third culture (square), closed symbols were used. For cases with four cultures (circle) or five cultures (triangle) needed to be able to detect VRE, open symbols were used. **b** Box-plot indicating the number of days between sample date of the first positive culture of the index patient and of the 51 secondary cases; median= 9, interquartile range (IQR) = 9

We determined whether the proxy parameter was linked to the rectal swab number of the first positive culture of a secondary case. When plotting the data by group (1^st^ until 5^th^ culture positive), we observed that a median of 9 days (interquartile range: 9) had passed after the positive culture of the index, before the secondary patient cultured positive (Fig. 3). With regards to the seven patients who were first cultured VRE-positive in the 4^th^ or 5^th^ culture, six could be analyzed. The number of days between the positive culture of the index patient and the first positive VRE culture of those six secondary cases were 9, 9, 10, 11, 16, and 31 days (Fig. 3).

## Discussion

The main finding of this study was that seven secondary cases (11%) would have been missed if only three rectal samples would have been obtained instead of five. Consequently, 89% of secondary cases of VRE transmission were identified in three consecutive rectal swab cultures.

Data on the number of cultures needed to detect VRE colonization after in-hospital transmission are so far limited. One previous study reported on the number of cultures needed to detect VRE transmission [13]. During an outbreak in Australia, 172 VRE carriers from one outbreak were analyzed. When three rectal swabs were acquired, 84% of carriers were detected. Four or five screening samples increased sensitivity to 92% and 95%, respectively, supporting our finding that an approach with more than three cultures leads to a higher VRE colonization detection rate.

Likewise, Frakking et al. reported on a large outbreak with three VRE clones, in which they found a detection rate of 95% after taking five consecutive rectal samples [14]. In contrast to this, Fonville et al. concluded that one negative PCR for *vanA* and *vanB* on a single rectum swab, after overnight incubation in enrichment broth, was sufficient to replace five screening cultures, even though they missed 1 out of 20 VRE-positive patients with this approach [15]. They, however, focused on the negative predictive value of qPCR compared to VRE culture with the purpose of reducing the pressure on isolation capacity. The authors have derived similar detection percentages of secondary cases in VRE contact tracing with a comparable screening protocol to ours (around 80% and 95% after four and five screening cultures, respectively; J. Fonville, personal communication).

Our current policy is to start screening for VRE transmission on the day a new VRE-positive patient is identified, to be able to define the extent of an outbreak early and be ahead of further transmission. This is under debate since colonization of a new host and subsequent detection by rectal swab culture might take some time. No clear data is published on the time to establish detectable colonization by VRE. Our study results indicate that day 9 after presumed exposure should be included in the contact tracing period and that starting contact tracing at a later time point might be useful. Furthermore, it could be hypothesized that the number of cultures needed to detect VRE transmission might then be reduced, although intermittent shedding would still be a relevant issue. There are some drawbacks in this scenario. First, our study estimated the moment of transmission by taking the day of VRE detection in the assumed index patients as moment of exposure, and only including *vanA*/*vanB* matches. Therefore, we hypothesize that the actual duration from the day of transmission from the index patient to the day of detection in a contact patient is longer than 9 days. Therefore, the 9 days is the minimum number of days. Also, by the above scenario, it takes a longer time to trace transmission, meaning that there would be more time for ongoing transmission, unless some IPC measures are taken for patients at risk. The need for more (isolation/cohort) rooms, more personal protective equipment and more personnel for contact precautions will lead to higher costs for appropriate healthcare, in addition to adverse effects on the patient’s wellbeing. Interestingly, the estimated median time needed for colonization appears quite long, although such data have to our knowledge not been published before. One of the explanations might be a sampling delay, since screening cultures were also obtained from patients who were already discharged at time of contact tracing. These patients received swabs per mail and were asked to take cultures and send these back to the hospital by mail. Sending out culture sets and sending back specimens takes more time than obtaining rectal swabs from in-hospital patients.

### Limitations

There are a few remarks concerning our study. First, the retrospective, single center nature of the analysis. Second, we only analyzed up to 5 screening samples, as is policy in our hospital based on the national guideline. Also, early in the study period obtaining five cultures was not routine yet, so secondary patients could have been missed. Third, our laboratory protocols for detection of VRE changed over the study period. Since sensitivity of screening could differ by applying other or newer methods, we analyzed using 2 pre-defined time periods (methods 1 and 2 compared to method 3 as defined in Online Resource 2) if the number of first positive VRE cultures changed over time; we could confirm that this did not change over time.

## Conclusion

In our setting, a fourth and fifth rectal screening culture added a considerable number of secondary VRE cases compared to screening with three rectal swabs. This increased the detection rate with at least 11%. Furthermore, our result show that one or more rectal swabs taken around day 9 after presumed exposure should at least be included in the screening approach. This implies that besides the number of swabs that are taken, also the timing of swabs after presumed exposure is of importance. Our results may give guidance on changing guidelines with respect to the most optimal timing and number of screening cultures. This will lead to a more efficient VRE contact screening procedure. However, it is preferable that these results will be confirmed in a larger (multicenter) setting.

## Supplementary Information

ESM 1 (PDF 660 KB)

ESM 2 (PDF 673 KB)

ESM 3 (PDF 660 KB)

ESM 4 (PDF 735 KB)

## Data Availability

The datasets generated during and/or analyzed during the current study are not publicly available due to ethical considerations but are available from the corresponding author on reasonable request.

## References

[1] Zhou X, Willems RJL, Friedrich AW, Rossen JWA, Bathoorn E (2020). *Enterococcus faecium*: from microbiological insights to practical recommendations for infection control and diagnostics. Antimicrob Resist Infect Control.

[2] Ulrich N, Vonberg RP, Gastmeier P (2017). Outbreaks caused by vancomycin-resistant *Enterococcus faecium* in hematology and oncology departments: a systematic review. Heliyon.

[3] Alevizakos M, Gaitanidis A, Nasioudis D, Tori K, Flokas ME, Mylonakis E (2017). Colonization with vancomycin-resistant enterococci and risk for bloodstream infection among patients with malignancy: a systematic review and meta-analysis. Open Forum. Infect Dis.

[4] Ruhnke M, Arnold R, Gastmeier P (2014). Infection control issues in patients with haematological malignancies in the era of multidrug-resistant bacteria. Lancet Oncol.

[5] Pan SC, Wang JT, Chen YC, Chang YY, Chen ML, Chang SC (2012). Incidence of and risk factors for infection or colonization of vancomycin-resistant enterococci in patients in the intensive care unit. PLoS One.

[6] Papadimitriou-Olivgeris M, Drougka E, Fligou F, Kolonitsiou F, Liakopoulos A, Dodou V, Anastassiou ED, Petinaki E, Marangos M, Filos KS, Spiliopoulou I (2014). Risk factors for enterococcal infection and colonization by vancomycin-resistant enterococci in critically ill patients. Infection.

[7] Kaki R, Yu Y, O'Neill C, Lee C, Mertz D, Control T, Hamilton Health Sciences Infection P (2014). Vancomycin-resistant enterococcus (VRE) transmission and risk factors in contacts of VRE carriers. Infect Control Hosp Epidemiol.

[8] D'Agata EM, Gautam S, Green WK, Tang YW (2002). High rate of false-negative results of the rectal swab culture method in detection of gastrointestinal colonization with vancomycin-resistant enterococci. Clin Infect Dis.

[9] Pearman JW, Perry PL, Kosaras FP, Douglas CR, Lee RC, Peterson AM, Orrell CT, Khinsoe CH, Heath CH, Christiansen KJ (2003). Screening and electronic labelling of ward contacts of vancomycin-resistant *Enterococcus faecium* vanB carriers during a single-strain hospital outbreak and after discharge from hospital. Commun Dis Intell Q Rep.

[10] Netherlands Society for Medical Microbiology (NVMM) (2015). NVMM guideline highly resistant microorganisms (HRMO): VRE.

[11] Weterings V, van Oosten A, Nieuwkoop E, Nelson J, Voss A, Wintermans B, van Lieshout J, Kluytmans J, Veenemans J (2021). Management of a hospital-wide vancomycin-resistant *Enterococcus faecium* outbreak in a Dutch general hospital, 2014-2017: successful control using a restrictive screening strategy. Antimicrob Resist Infect Control.

[12] de Smet AM, Kluytmans JA, Blok HE, Mascini EM, Benus RF, Bernards AT, Kuijper EJ, Leverstein-van Hall MA, Jansz AR, de Jongh BM, van Asselt GJ, Frenay IH, Thijsen SF, Conijn SN, Kaan JA, Arends JP, Sturm PD, Bootsma MC, Bonten MJ (2011). Selective digestive tract decontamination and selective oropharyngeal decontamination and antibiotic resistance in patients in intensive-care units: an open-label, clustered group-randomised, crossover study. Lancet Infect Dis.

[13] Pearman JW (2006). 2004 Lowbury Lecture: the Western Australian experience with vancomycin-resistant enterococci - from disaster to ongoing control. J Hosp Infect.

[14] Frakking FNJ, Bril WS, Sinnige JC, Klooster JEV, de Jong BAW, van Hannen EJ, Tersmette M (2018). Recommendations for the successful control of a large outbreak of vancomycin-resistant *Enterococcus faecium* in a non-endemic hospital setting. J Hosp Infect.

[15] Fonville JM, van Herk CMC, Das P, van de Bovenkamp JHB, van Dommelen L (2017). A single negative result for van quantitative PCR on enrichment broth can replace five rectal swab cultures in screening for vancomycin-resistant enterococci. J Clin Microbiol.

