# Supplementary material for: Contact tracing for vancomycin-resistant Enterococcus faecium (VRE): evaluation of the Dutch policy of quintuple screening cultures
Source: Eur J Clin Microbiol Infect Dis. 2023 Jun 23;42(8):993–9. doi: 10.1007/s10096-023-04632-7 (PMC10345005; doi:10.1007/s10096-023-04632-7)
Supplement: Supplementary file 1 — (PDF 660 KB) [file 10096_2023_4632_MOESM1_ESM.pdf]

Contact tracing for vancomycin-resistant *Enterococcus faecium* (VRE): evaluation of the Dutch policy of quintuple screening cultures

Linda J. Wammes MD PhD[a$#], Anne F. Voor in ’t holt PhD[a#], Corné H.W. Klaassen PhD[a], Margreet C. Vos MD PhD[a], Nelianne J. Verkaik MD PhD[a], Juliëtte A. Severin MD PhD[a]*

[a]Department of Medical Microbiology and Infectious Diseases, Erasmus MC University Medical Center Rotterdam, P.O. Box 2040, 3000 CA, The Netherlands

[$]Present address: Department of Medical Microbiology, LUMC Center for Infectious Diseases, Leiden University Medical Center, Leiden, The Netherlands

[#] shared first authorship

*Corresponding author: Dr. Juliëtte Severin. Department of Medical Microbiology and Infectious Diseases, Erasmus MC University Medical Center, P.O. Box 2040, 3000 CA, Rotterdam, The Netherlands. E-mail: j.severin@erasmusmc.nl. Telephone: +31 10 703 28 79.

Supplementary table 1. Methods for contact tracing during the study period (2010 – 2018).

Period	Contact tracing indication	Contact tracing method
**March 18, 2010[1], until November 25, 2012**	1. N≥2 patients identified with VRE at the same department, and suspected transmission.<br>2. Patient population:<br>- Patients admitted to the ICU.<br>- Patients with haematological disorders.<br>- Patients undergoing haemodialysis.<br>Other departments/patient population only on indication.	1. Were identified VRE patients admitted at the same room?<br>a. Screen current roommates.<br>b. Screen former roommates who are still admitted.<br>2. Were identified VRE patients not admitted at the same room?<br>a. Screen all patients still admitted at the department.<br>b. Screen all ward mates still admitted but transferred to other wards.<br>c. Screen patients transferred to other healthcare facilities.<br>Discharged patients are outside of the scope of the contact tracing.
**November 26, 2012, until July 17, 2013**	1. N≥2 patients identified with VRE at the same department (irrespective of the department), and suspected transmission.<br>2. In exceptional cases N=1 is sufficient to start contact tracing.	1. Were identified VRE patients admitted at the same room?<br>a. Screen current roommates<br>b. Screen former roommates who are still admitted.<br>2. Were identified VRE patients not admitted at the same room?<br>a. Screen all patients still admitted at the department<br>b. Screen all ward mates still admitted but transferred to other wards<br>c. Screen patients transferred to other healthcare facilities<br>Discharged patients are outside of the scope of the contact tracing.
**July 18, 2013, until October 2, 2013**	N=1 if unexpected finding without adequate IPC measures (i.e., including isolation).	1. Screen all current and former room and ward mates who are still admitted<br>2. Contact all former room and ward mates who are already discharged and ask them to screen by sending a culture set by mail.
**October 3, 2013, until end of study period (i.e., January 2018)**	N=1 if unexpected finding without adequate IPC measures (i.e., including isolation).	1. Screen all current and former ward mates still admitted<br>2. Contact all former room and ward mates who are already discharged and ask them to screen by sending a culture set by mail.<br>3. Current and former roommates will be given a 'suspected VRE' label in the EHR and will be cared for in isolation upon admittance until screened negative.

Abbreviations: EHR; Electronic Health Record, ICU; Intensive Care Unit, IPC; Infection Prevention and Control measures, N; number, VRE; vancomycin-resistant *Enterococcus faecium*.

[1] March 18, 2010 was the publication of the first guideline which included contact tracing methods for VRE.
