# Supplementary material for: Contact tracing for vancomycin-resistant Enterococcus faecium (VRE): evaluation of the Dutch policy of quintuple screening cultures
Source: Eur J Clin Microbiol Infect Dis. 2023 Jun 23;42(8):993–9. doi: 10.1007/s10096-023-04632-7 (PMC10345005; doi:10.1007/s10096-023-04632-7)
Supplement: Supplementary file 2 — (PDF 673 KB) [file 10096_2023_4632_MOESM2_ESM.pdf]

Contact tracing for vancomycin-resistant *Enterococcus faecium* (VRE): evaluation of the Dutch policy of quintuple screening cultures

Linda J. Wammes MD PhD[a$#], Anne F. Voor in ’t holt PhD[a#], Corné H.W. Klaassen PhD[a], Margreet C. Vos MD PhD[a], Nelianne J. Verkaik MD PhD[a], Juliëtte A. Severin MD PhD[a*]

[a]Department of Medical Microbiology and Infectious Diseases, Erasmus MC University Medical Center Rotterdam, P.O. Box 2040, 3000 CA, The Netherlands

[$]Present address: Department of Medical Microbiology, LUMC Center for Infectious Diseases, Leiden University Medical Center, Leiden, The Netherlands

[#] shared first authorship

*Corresponding author: Dr. Juliëtte Severin. Department of Medical Microbiology and Infectious Diseases, Erasmus MC University Medical Center, P.O. Box 2040, 3000 CA, Rotterdam, The Netherlands. E-mail: j.severin@erasmusmc.nl. Telephone: +31 10 703 28 79.

Supplementary table 2. Methods for vancomycin-resistant *Enterococcus faecium* (VRE) screening during the study period (2010 – 2018).

	**Method 1**	**Method 2**	**Method 3**
**Time period**	2010 – 2012	2013 – 2014	2015 – 2018
**Broth**	Phenol mannitol broth[1] + 6 mg/L vancomycin	Phenol mannitol broth[1] + 6 mg/L vancomycin	Enterococcosel broth[2] + 8 mg/L amoxicillin
**Incubation time**	2x overnight	2x overnight	1x overnight
**Method on broth**	Direct culture	rt-PCR<br>targets: *vanA* + *vanB*<br>culture when positive	rt-PCR<br>targets: *vanA* + *vanB* + *recG* (*E. faecium*)<br>culture when positive
**Type of agar**	Blood agar[2]	Blood agar[2]	Selective agar with vancomycin[3]<br>(± blood agar[$])
**Identification of *Enterococcus faecium***	Vitek[4]	MALDI-TOF[5]	MALDI-TOF[5]
**Susceptibility testing**	Vitek[4] +<br>E-tests vancomycin, teicoplanin	Vitek[4] +<br>E-tests vancomycin, teicoplanin	Vitek[4] +<br>E-tests vancomycin, teicoplanin
**Genotypic confirmation**	Conventional PCR for *vanA* + *vanB* + *vanC*	rt-PCR for *vanA* + *vanB*	GeneXpert[6] for *vanA* + *vanB*

[1]BBL, Le Pont de Claix, France; [2]Becton Dickinson, Breda, the Netherlands; [3]Brilliance™ VRE agar, Oxoid, ThermoScientific, Waltham, USA; [4]Vitek®2, bioMérieux, Marcy-l'Etoile, France; [5]Bruker, Karlsruhe, Germany; [6]Cepheid, Sunnyvale, USA

[$]positive broth was also inoculated on an additional blood agar when rt-PCR was strongly positive (cycle threshold value <30) for *recG* and *vanB* with the purpose of screening for *vanB*-positive VRE with low MIC for vancomycin

Method 1 (2010-2012):

A cotton swab (Amies medium; Becton Dickinson, Breda, The Netherlands) was placed in 5 mL phenol mannitol broth (BBL, Le Pont de Claix, France) containing 6 mg/L vancomycin and incubated for two overnights at 35°C. Subsequently, a loopful of broth was subcultured on a Colombia blood agar plate (BD) for overnight incubation at 35°C. Colonies suspect for enterococci were further analyzed by Vitek®2 (bioMérieux) for identification and antimicrobial susceptibility testing, vancomycin and teicoplanin E-tests (bioMérieux), and in case of resistance to one of the glycopeptides, conventional PCR for *vanA, vanB* and *vanC* genes performed in-house and confirmed at the Utrecht University Medical Center (UMCU).

Method 2 (2013-2014):

A cotton swab (Amies medium) was placed in 5 mL phenol mannitol broth (BBL, Le Pont de Claix, France) containing 6 mg/L vancomycin and incubated for two overnights at 35°C. After incubation, a multiplex real-time PCR targeting *vanA* and *vanB* genes was performed on the broth. In case of a positive PCR, the broth was subcultured on a blood agar plate. Colonies were further analyzed as described for method 1, except for the identification, for which MALDI-TOF (Bruker, Karlsruhe, Germany) was used instead of Vitek®2. Also, detection of *vanA* and *vanB* in suspected isolates was done using an in-house developed rt-PCR, and always confirmed by the UMCU laboratory.

Method 3 (2015 - 2018)

A cotton swab was placed in a 5 mL Enterococcosel broth (BD) containing 8 mg/L amoxicillin. After one overnight incubation at 35°C, 200 µL of broth was used for DNA isolation (Magna Pure 96 system), followed by a multiplex real-time PCR targeting *E. faecium* (*recG* gene), *vanA*, and *vanB*. In case of a positive PCR for the *E. faecium* target (Ct value ≥30) in combination with either *vanA* (any Ct-value) or *vanB* (Ct value ≥30), 10 µL of broth was subcultured on a selective agar with vancomycin (Brilliance™ VRE agar; Oxoid, ThermoScientific, Waltham, USA). In case of a positive PCR for the *E. faecium* target (Ct value <30) in combination with *vanB* (Ct-value <30), 10 µL of broth was subcultured on the selective agar and a blood agar plate – with the purpose of not missing VRE strains with low MIC for vancomycin. The Brilliance™ VRE agars were incubated for at least 48 hours according to the manufacturer's instructions. Suspect colonies were further analyzed as described in Method 2. However, the GenXpert (Cepheid) was used to detect *vanA* or *vanB* genes in isolates. Cg-MLST was performed on indication.
