# Supplementary material for: Contact tracing for vancomycin-resistant Enterococcus faecium (VRE): evaluation of the Dutch policy of quintuple screening cultures
Source: Eur J Clin Microbiol Infect Dis. 2023 Jun 23;42(8):993–9. doi: 10.1007/s10096-023-04632-7 (PMC10345005; doi:10.1007/s10096-023-04632-7)
Supplement: Supplementary file 3 — (PDF 660 KB) [file 10096_2023_4632_MOESM3_ESM.pdf]

Contact tracing for vancomycin-resistant *Enterococcus faecium* (VRE): evaluation of the Dutch policy of quintuple screening cultures

Linda J. Wammes MD PhD[a$#], Anne F. Voor in ’t holt PhD[a#], Corné H.W. Klaassen PhD[a], Margreet C. Vos MD PhD[a], Nelianne J. Verkaik MD PhD[a], Juliëtte A. Severin MD PhD[a]*

[a]Department of Medical Microbiology and Infectious Diseases, Erasmus MC University Medical Center Rotterdam, P.O. Box 2040, 3000 CA, The Netherlands

[$]Present address: Department of Medical Microbiology, LUMC Center for Infectious Diseases, Leiden University Medical Center, Leiden, The Netherlands

[#] shared first authorship

*Corresponding author: Dr. Juliëtte Severin. Department of Medical Microbiology and Infectious Diseases, Erasmus MC University Medical Center, P.O. Box 2040, 3000 CA, Rotterdam, The Netherlands. E-mail: j.severin@erasmusmc.nl. Telephone: +31 10 703 28 79.

Supplementary table 3. Hospital outbreaks with vancomycin-resistant *Enterococcus faecium* (VRE) during the study period.

VRE Outbreak	Main departments involved	Year	Total no. of positive VRE patients	VRE detection method (see Supplementary table 1)	No. of patients in contact investigation	Typing method
A (*vanA*)	Cardiothoracic surgery and ICU	2011	13	1	28 (round 1)<br>196 (round 2)	Raman spectroscopy
B (*vanB*)	Gastroenterology	2012-2013	8	2	471	Raman spectroscopy, MLST and cg-MLST (ST117; cg-MLST 24)
C (*vanA*)	Pediatrics	2014	4	2	±400	Raman spectroscopy, MLST and cg-MLST (ST18, and ST117; cg-MLST 24)
D (*vanB*)	Surgery, neurosurgery, and gastroenterology	2015	33	3	1532	Raman spectroscopy, cg-MLST (cg-MLST 103 mainly)
E (*vanA*)	Multiple	2015-2016	28	3	453	Raman spectroscopy, cg-MLST (cg-MLST 20 mainly)
F (*vanA*)	Hematology	2016	3	3	48	cg-MLST (ST80, cg-MLST 1070)
G (*vanA*)	ICU and neurosurgery	2016	3	3	52	cg-MLST (ST80, cg-MLST 106)
H (*vanB*)	Neurosurgery	2016	3	3	99	cg-MLST (cg-MLST 103)
I (*vanA*)	Obstetrics	2016	2	3	52	cg-MLST (cg-MLST 1071)

Abbreviations: ICU; intensive care unit, no.; number of, VRE; vancomycin-resistant *Enterococcus faecium*, MLST; multi-locus sequence typing, cg-MLST; core-genome MLST, ST; sequence type.
