# Supplementary material for: Contact tracing for vancomycin-resistant Enterococcus faecium (VRE): evaluation of the Dutch policy of quintuple screening cultures
Source: Eur J Clin Microbiol Infect Dis. 2023 Jun 23;42(8):993–9. doi: 10.1007/s10096-023-04632-7 (PMC10345005; doi:10.1007/s10096-023-04632-7)
Supplement: Supplementary file 4 — (PDF 735 KB) [file 10096_2023_4632_MOESM4_ESM.pdf]

Contact tracing for vancomycin-resistant *Enterococcus faecium* (VRE): evaluation of the Dutch policy of quintuple screening cultures

Linda J. Wammes MD PhD[a$#], Anne F. Voor in 't holt PhD[a#], Corné H.W. Klaassen PhD[a], Margreet C. Vos MD PhD[a], Nelianne J. Verkaik MD PhD[a], Juliëtte A. Severin MD PhD[a]*

[a]Department of Medical Microbiology and Infectious Diseases, Erasmus MC University Medical Center Rotterdam, P.O. Box 2040, 3000 CA, The Netherlands

[$]Present address: Department of Medical Microbiology, LUMC Center for Infectious Diseases, Leiden University Medical Center, Leiden, The Netherlands

[#] shared first authorship

*Corresponding author: Dr. Juliëtte Severin. Department of Medical Microbiology and Infectious Diseases, Erasmus MC University Medical Center, P.O. Box 2040, 3000 CA, Rotterdam, The Netherlands. E-mail: j.severin@erasmusmc.nl. Telephone: +31 10 703 28 79.

Supplementary table 4. Results of vancomycin-resistant *Enterococcus faecium* (VRE) screening cultures from the 61 included patients.

Patient	Screening culture 1	Screening culture 2	Screening culture 3	Screening culture 4	Screening culture 5	Screening culture 6	Screening culture 7	Screening culture 8
**1 screening culture available**								
**1**	pos							
**2**	pos							
**3**	pos							
**4**	pos							
**5**	pos							
**6**	pos							
**7**	pos							
**2 screening cultures available**								
**8**	pos	pos						
**9**	pos	pos						
**10**	pos	pos						
**11**	neg	pos						
**12**	neg	pos						
**3 screening cultures available**								
**13**	neg	neg	pos					
**4 screening cultures available**								
**14**	pos	pos	neg	pos				
**15**	pos	pos	neg	neg				
**16**	pos	pos	pos	neg				
**17**	pos	pos	pos	pos				
**18**	neg	pos	pos	neg				
**19**	neg	neg	pos	pos				
**20**	neg	neg	neg	pos				
**5 screening cultures available**								

**21**	pos	pos	pos	pos	pos			
**22**	pos	pos	pos	pos	pos			
**23**	pos	pos	pos	pos	pos			
**24**	pos	pos	pos	pos	pos			
**25**	pos	pos	pos	pos	pos			
**26**	pos	pos	pos	pos	pos			
**27**	pos	pos	pos	pos	pos			
**28**	pos	pos	pos	pos	pos			
**29**	pos	pos	pos	pos	pos			
**30**	pos	pos	pos	pos	pos			
**31**	pos	pos	pos	pos	pos			
**32**	pos	pos	pos	pos	neg			
**33**	pos	pos	neg	pos	neg			
**34**	pos	pos	neg	pos	pos			
**35**	pos	neg	pos	neg	neg			
**36**	pos	neg	neg	neg	neg			
**37**	neg	pos	pos	neg	neg			
**38**	neg	pos	neg	pos	neg			
**39**	neg	pos	neg	pos	neg			
**40**	neg	pos	neg	pos	neg			
**41**	neg	neg	pos	pos	pos			
**42**	neg	neg	pos	pos	pos			
**43**	neg	neg	pos	pos	neg			
**44**	neg	neg	pos	neg	neg			
**45**	neg	neg	pos	neg	neg			
**46**	neg	neg	pos	neg	neg			
**47**	neg	neg	neg	pos	pos			
**48**	neg	neg	neg	pos	neg			
**49**	neg	neg	neg	pos	neg			

**50**	neg	neg	neg	pos	pos			
**51**	neg	neg	neg	neg	pos			
**52**	neg	neg	neg	neg	pos			
**>5 screening cultures available**								
**53**	pos	pos	pos	pos	pos	pos		
**54**	pos	pos	pos	pos	pos	neg		
**55**	pos	pos	pos	pos	pos	neg		
**56**	pos	pos	neg	pos	neg	neg		
**57**	pos	pos	neg	neg	neg	neg		
**58**	pos	neg	neg	pos	neg	neg		
**59**	pos	neg	neg	neg	neg	neg		
**60**	pos	neg	neg	neg	neg	neg		
**61**	pos	pos	neg	pos	pos	pos	pos	pos

Abbreviations: neg; negative VRE culture, pos; positive VRE culture, VRE; vancomycin-resistant *Enterococcus faecium*.
